# How and When to Deliver the Placenta

**Published:** 1885-04-20

**Authors:** H. B. Ritter

**Affiliations:** Adjunct Professor of Obstetrics and Gynecology in the Louisville Medical College


### HOW AND WHEN TO DELIVER THE PLACENTA.

By H. B. Ritter, M. D.,

**Adjunct Professor of Obstetrics and Gynecology in the Louisville Medical College.**

There is no subject in obstetrics on which more has been written than on the delivery of the placenta, and none, perhaps, on which there is less unity of opinion. This diversity on a subject so practical, one that we witness so frequently, and of which the physiology is so well understood, is hardly to be accounted for. As so much has already been written we will scarcely expect to say anything entirely new, but will treat the subject as it appears to us from a physiological and practical study.

To begin with, we believe that the third stage of labor should be treated on the same general principles as the first and second stages. We say this because normally the action of the uterus is the same in the third stage as in the first and second, and also because the departures from the normal action of the uterus are alike in the three stages.

In the third stage there is naturally a tonic and spasmodic contraction of the uterus, the functions of which are to condense the uterus, to separate the placenta and cause its expulsion. In the first and second stages these same contractions are present to condense the uterus and prevent complete recession of the presenting part, and cause expulsion of the child.

When we look at the abnormalties attending the delivery of the placenta we find them duplicates of those attending the first and second stages. For instance, we find irregular contraction of the uterus in the third stage, usually as the familiar hour-glass contraction. This is found in the first stages as contraction of the fibres around the internal os, preventing dilatation, and also as "ante-partum hour-glass contraction." Again, every one knows that either inertia or excessive contractions may attend either of the three stages. These abnoimal conditions are constantly assuming differeut phases, but in the main they are alike. Now, if the uterus presents the same forms, normal and abnormal, in the three stages, and as the object of the uterus from the beginning of the first stage to the end of the third stage is to expel its contents and to condense itself, there is no reason why the principles of treatment should be changed when two of the stages are completed. There is no reason for a wider difference of opinion on the management of the third stage than on the two preceding. Because the third stage can be terminated at the will of the attendant with very little trouble is no justifiable reason for so doing. It might be argued from the same standpoint that in the first and second stages delivery should be hastenened by artificial dilation of the neck of the uterus, turning, and the use of forceps. Certainly no one would advise such measures unless there was some complication which demanded them. We are to resort to these methods only after nature is found inefficient to do her work and the welfare of the patient is endangered by further delay. So with reference to the management of the third stage.

In natural cases, to co-operate with nature, we should not interfere during the first fifteen or twenty minutes after the birth of the child. This is a period of repose, during which we should expect nothing but a firm and regular tonic contraction of the uterus. It is our duty, however, to assure ourselves that this natural contraction exists, by now and then grasping the uterus through the abdominal wall, but make no friction, nor knead it unless the tonic contraction is deficient. If with the want of tonic contraction found at this time there is hemorrhage the indications are to deliver the placenta at once by introducing the hand into the uterus, if kneading and pressing on the fundus have failed.

If the spasmodic contractions come on in fifteen or twenty minutes and are sufficiently strong, we follow the same course as with similar pains in either of the preceding stages. Let them alone, but place the hand over the uterus to keep us informed. It happens frequently, however, that these pains are too feeble, and then we are called upon to assist. Here pressure with the palm of the hand on the fundus, and in a direction downward and backward will increase the contraction to a natural one and express the placenta, and this is the proper treatment. Again, when the spasmodic contractions fail to come on in the usual time, this pressure should be made at intervals to imitate nature and supply that element which is missing. In irregular contraction of the uterus when not attended with hemorrhage, speedy delivery is not indicated. The placenta will come away, and the forcing of the hand through the constriction be avoided. In this connection I will relate a recent experience as an illustration. The child was born about ten minutes before my arrival. The uterus was found irregularly contracted and reached to the lower border of the ribs on the right side. There was no hemorrhage, and therefore no immediate danger. I concluded to wait and to watch, leaving all to nature. Spasmodic contractions soon came on and were rather strong, but the placenta was not expelled at once. During one of the contractions, just one hour from the termination of the second stage, the uterus rounded up and the placenta was expelled, and with it not even as much blood as is usual in normal cases. Suppose that we had terminated the third stage by removing the placenta with the hand carried into the womb. There would necessarily have been hemorrhage, and we would have run the risk of injuring the tissues of the uterus by forcing the hand through the constricted part. In these cases we believe it sufficient time to act when hemorrhage once begins, or some other indication for delivery manifests itself.

There is a time in all cases when, if the placenta is retained, it should be removed. But as we cannot say to deliver with forceps if the child is not born in so many hours, so in the third stage there is no fixed time for delivering the placenta. Like the first and second stages, it should not be allowed to continue too long. It is certainly very exhausting to a woman to be worried by recurring pains and the knowledge that labor is not completed, and she therefore not out of danger. We must therefore take into consideration her general condition as well as the kind and force of uterine contractions to determine when the placenta should be removed. In some cases this will be in fifteen or twenty minutes; in others not until forty or sixty minutes.

Those that have adopted the expectant method of treatment are certainly extremists and wait entirely too long in many cases, allowing patients to become worn out and exhausted. Prolongation of the third stage is frequently found, it should be remembered, in debilitated and enfeebled states of the system.

Those who adopt Crede's method go to the other extreme of not waiting long enough, and outwitting nature. We are told, that with the expectant method delivery occurs in nearly all cases within two hours, while Crede terminates the majority of his cases in four or five minutes. Crede therefore dispenses with the period of repose which naturally follows after the birth of the child, and during which nature separates the placenta from the uterus and allows coagulas to form in the uterine sinues. We cannot believe it proper treatment to apply the whip to the uterus simply because it will respond.

Traction on the cord has deservedly fallen into disrepute. We look upon this as a poor method of delivery under all circumstances, because a better can be used, to say nothing of the irregular contraction and inversion of the uterus that may be produced by it.

Ergot is sometimes indispensable in the third stage. It does not produce the kind of contraction wanted to expel the placenta and sometimes prevents expulsion.

The method of the Dublin school is more according to nature than any other method of conducting the delivery of the placenta. When the spasmodic contractions are feeble we adopt this plan. Guard the uterus by means of the hand over the fundus, and when a pain comes on make a pressure to assist in the expulsion. When the contractions are strong we are not in favor of their pressure because nature makes all the pressure that is necessary. It will also be noticed that patients generally complain of pressure at such times as being very painful.—*The Medical Herald.*

